# Antinociceptive and neuropharmacological activities of methanol extract of *Phoenix sylvestris* fruit pulp

**DOI:** 10.3389/fphar.2015.00212

**Published:** 2015-10-02

**Authors:** Md. Shafiullah Shajib, Saleha Akter, Tajnin Ahmed, Mohammad Zafar Imam

**Affiliations:** ^1^Department of Pharmacy, Stamford University BangladeshDhaka, Bangladesh; ^2^Department of Pharmacy, Primeasia UniversityDhaka, Bangladesh

**Keywords:** *Phoenix sylvestris* Roxb., Arecaceae, ethnomedicine, antinociceptive, CNS depressant, sedative, anxiolytic

## Abstract

Fruits of *Phoenix sylvestris* Roxb. (Arecaceae) are used to treat back pain, toothache, headache, arthritis, nervous debility and as sedative. The aim of this study was to evaluate the antinociceptive and neuropharmacological activities of methanol extract of *P. sylvestris* fruit pulp (MEPS). The antinociceptive activity of MEPS was evaluated by heat-induced (hot plate, tail immersion test) and chemical-induced pain models (acetic acid-induced writhing, formalin-induced nociception, glutamate-induced nociception and paw edema test). The effect of MEPS on central nervous system (CNS) was studied using hole cross test, open field test, sodium thiopental-induced sleeping time and elevated plus maze test. MEPS showed strong, significant and dose-dependent antinociceptive activity in all heat-induced and chemical-induced pain models at all experimental doses. Involvement of opioid receptor mediated analgesia was evident from the reversal of analgesic effect by naloxone. MEPS also showed reduced locomotor activity in both hole cross and open field tests. The increase in sleeping time in sodium thiopental-induced sleeping test and anxiolytic activity in elevated plus maze test were also significant. So, it is evident that MEPS possesses strong central and peripheral antinociceptive activity as well as CNS depressant, sedative and anxiolytic activity. The results justify the ethnomedicinal use of *P. sylvestris* fruit in different painful conditions and CNS disorders.

## Introduction

Investigation of pharmacological basis of the therapeutic uses of plants by traditional practitioners is an integral part of ethnopharmacology (Leonti and Casu, 2013). Ethnopharmacological knowledge continues to contribute to the discovery of new antinociceptive agents from plants (Calixto et al., 2000). Phytotherapy based on this knowledge is also being used as a guide for the development of CNS depressant, sedative, anxiolytic drugs (Gurib-Fakim, 2006).

*Phoenix sylvestris* Roxb. (Arecaceae), locally known as Khejur, is a palm tree cultivated for its syrupy juice and edible fruit in Bangladesh. Fruits of the plant are used to treat back pain, stomachache, toothache, headache, arthritis, pain of buttocks, fever, piles, nervous debility, and as nervine tonic, restorative, sedative in ethnomedicine (Ghani, 2003; Acharya and Pokhrel, 2006; Harney, 2013; Manikandan, 2013). Besides, fruits are used in cough, diarrhea, dysentery, opthalmia, opacity of cornea, and toothworm (Ghani, 2003; Ahmad et al., 2008; Rajkumari et al., 2013; Murthy and Madhav, 2015). Fruits are also employed as aphrodisiac, cooling and gastric stimulant in traditional medicine (Goswami et al., 2012; Sambandan and Dhatchanamoorthy, 2012). Amino acids (mainly alanine), vitamins A, B, and D, sugars, tannins, mucilage and ascorbic acid have been found in fruits (Ghani, 2003). Proanthocyanidin, a polyphenolic substance belonging to the flavonoid group, has been isolated from the fruits of *P. sylvestris* (Rao et al., 1980). The antioxidant activity of fruits has been reported (Mukherjee et al., 2014). Aqueous extract of the fruits has been found to inhib the angiotensin I-converting enzyme (ACE), α-glucosidase and α-amylase (Das et al., 2012; Das and De, 2013). The erythropoietic activity of the fruits has also been reported (Lohar et al., 2009).

Ethnomedicinal reports on *P. sylvestris* fruits demonstrate its extensive application in different painful conditions and neurological disorders. However, there is lack of scientific study regarding antinociceptive as well as neuropharmacological activities of *P. sylvestris* fruits. Therefore, we aimed to investigate the central and peripheral antinociceptive activities of *P. sylvestris* fruit pulp using heat-induced (hot plate and tail immersion test) and chemical-induced pain models (acetic acid-induced writhing, formalin induced-licking and glutamate-induced paw licking test). For the evaluation of neuropharmacological activities we studied locomotor activity by open field and hole cross method, sedative activity by sodium thiopental-induced sleeping time test and anxiolytic activity by elevated plus maze test. In addition, we have also reported the bioactive compounds by gas chromatography-mass spectroscopy (GC-MS) analysis of *P. sylvestris* fruit pulp.

## Materials and methods

### Collection, identification and extraction of plant material

Mature but unripe fruits of *P. sylvestris* were collected from Akabpur, Mainamati, Comilla, Bangladesh in July 23, 2013. Fruits were identified by Sarder Nasir Uddin, Senior Scientific Officer, Bangladesh National Herbarium, Mirpur, Dhaka, Bangladesh. A voucher specimen (DACB: 38499) has been deposited in the herbarium for further reference. The pulp of the fruits was separated from pit, dried and grounded. The powdered pulp (230 g) was macerated using 1000 mL of methanol with occasional stirring at 25 ± 2°C for 7 days. The extract was then filtered using sterilized cotton filter and Buchner funnel. A rotary evaporator was used to remove the solvent at 40° C and 50 r.p.m. under reduced pressure. After removal of solvent, 30.19 g (yield 13.13 %) dried extract was obtained and this crude extract was used for the experiments.

### Ethical statements

Ethical Principles and Guidelines of Scientific Experiments on Animals (1995) formulated by The Swiss Academy of Medical sciences and the Swiss Academy of Sciences were followed for the care and treatment of the animals. All the procedures and protocols were approved by the Ethics Committee of Stamford University Bangladesh (SUB/IAEC/13.02). The animals were euthanized using pentobarbital in accordance with AVMA guidelines for the Euthanasia of Animals: 2013 edition and sufficient measures were taken to minimize suffering prior to, during and after the experiments.

### Animals

Swiss albino mice were collected from Animal Resources Branch of International Centre for Diarrhoeal Disease Research, Bangladesh (icddr,b). The animals were housed in 120 × 30 × 30 cm cages and flake wood shavings were provided for bedding. The health status of mice was checked by daily monitoring during acclimatization period and before and after experiments. The animals were provided with standard diet and tap water *ad libitum*. For the period of 14 days animals were acclimatized in the laboratory environment before experiments. The animals were fasted overnight only before experiments. The standard laboratory conditions (relative humidity 55–60%; room temperature 25 ± 2°C; 12 h light/dark cycle with lights on at 7:00 a.m. and off at 7:00 p.m.) were maintained. Animals weighing between 25–30 g and 8–10 weeks aged were used for the experiments. All the experiments were performed between 9:00 a.m. and 5.00 p.m.

### Drugs and chemicals used in the experiments

The drugs and chemicals used in this study are: diclofenac sodium (Novartis Bangladesh Ltd.), morphine sulfate, sodium thiopental (Gonoshasthaya Pharmaceuticals Ltd.), diazepam (Square Pharmaceuticals Ltd.), naloxone hydrochloride (Samarth Life Sciences Pvt. Ltd., India), methanol, acetic acid, formalin, L-glutamic acid (Merck, Germany).

### Standard drugs and treatments

Physiological saline (0.9% sodium chloride) was used for the preparation of all the standard drugs and MEPS doses. It was used as vehicle at the dose of 10 mL/kg for the control groups. The doses (50, 150, 300, and 450 mg/kg body weight) of MEPS were selected on the basis of the effects of the trial dose and previously reported effective analgesic doses of the methanol extract of *P. sylvestris* (Howlader et al., 2006). The vehicle and MEPS were administered per oral (p.o) 30 min before the experiments. Morphine sulfate and diclofenac sodium were used as standard drug at the dose of 5 mg/kg and 10 mg/kg respectively in antinociceptive tests. Both of them were administered intraperitoneally (i.p.) 15 min before the experiments. In hot plate and tail immersion test naloxone was employed (i.p.) 15 min before the administration of morphine sulfate or MEPS, at the dose of 2 mg/kg to evaluate opioid mediated antinociceptive activity. Diazepam was used as standard drug in open field, hole cross, sodium thiopental-induced sleeping time and elevated plus maze test at the dose of 1 mg/kg (i.p.). In sodium thiopental-induced sleeping time and elevated plus maze test diazepam was administered 30 min prior to starting experiments. Sodium thiopental was administered (i.p.) at the dose of 40 mg/kg.

### Preliminary phytochemical screening

The crude extract of *P. sylvestris* fruits was qualitatively tested for phythochemicals using following reagents and chemicals: Carbohydrates with Molisch's and Fehling's reagents, reducing sugars with Benedict's reagent, glycosides with aqueous NaOH, glucosides with Fehling's reagent and H_2_SO_4_, resins with acetic anhydride and H_2_SO_4_, tannins with Ferric chloride and Lead acetate, proteins with NaOH and Cu_2_SO_4_, saponins with stable foam producing ability, flavonoids with the use of Zn and HCl, alkaloids with Mayer's, Hager's, Wagner's and Dragendroff's reagents and steroids with Libermann-Burchard's reagent. The change of color in respective tests was observed according to the standard procedures to detect the chemical constituents (Ghani, 2005).

### GC-MS (gas chromatography-mass spectroscopy) analysis

The GC-MS analysis was performed using Agilent Technologies 7890A capillary gas chromatograph, directly coupled to a mass spectrometer system (Model: 5975C inert XL EI/CI MSD with triple axis detector). A fused silica capillary column of 5% phenyl, 95% dimethyl-poly-silloxane (HP-5MSI; length: 90 m, diameter: 0.250 mm and film: 0.25 μm) was used. The GC parameter was set as follows: the inlet temperature was set at 250°C and oven temperature was programmed as 90°C for 0 min, then 3°C/min to 200°C for 2 min and then 15°C/min to 280°C for 2 min. Total run time was 46 min and column flow rate was 1.1 mL/min Helium gas. The auxiliary (GC to MS interface) temperature was set to 280°C. The MS parameter was set as the MS was in scan mode. The ionization mode was EI (electron ionization) type. The mass range was set in the range of 50–550 m/z. MS quad temperature and source temperature was set at 150°C and 230°C respectively. Each component were searched and identified by using “NIST-MS Library 2009.” Peak area of the total ionic chromatogram (TIC) was used to determine the relative percentage amounts of separated compounds and calculation were done automatically.

### Acute toxicity test

The acute toxicity of MEPS was observed in seven experimental groups. Each group contained five animals (*n* = 5). The experimental groups received MEPS at the dose of 500, 1000, 2000, 3000, 4000, 5000, and 6000 mg/kg body weight (p.o). Each group of animals were placed in separate cages and allowed for free access of water *ad libitum* and food. The animals were observed for the next 72 h to find any mortality, adverse reactions like skin rashes, itching, swelling and behavioral changes (Walker et al., 2008).

### Experimental procedures for antinociceptive test

#### Hot plate test

Hot plate test was performed using Eddy's hot plate (Kshitij Innovations, Haryana, India) for determining the central analgesic activity of MEPS. The temperature of hot plate was maintained at 55 ± 1°C. A pre-treatment latency was recorded as baseline for the study. Acute responses like jumping, forepaw licking and withdrawal of paw(s) were considered as nociception. After the treatment of mice with vehicle, MEPS (p.o) or standard drug morphine (i.p.) the latency periods were recorded at 30, 45, 60, 90, and 120 min (Eddy and Leimbach, 1953). The mice were placed in hot plate not more than for 20 s to avoid any tissue damage. The procedure was repeated by employing naloxone (i.p.) 15 min before morphine sulfate or MEPS administration in mice for the evaluation of opioid receptor involvement and the data were pooled. To determine the percentage of maximal possible effect (% MPE) following formula was used (Coelho et al., 2005):

$$\%\text{ MPE = }\frac{\text{Postdrug latency }-\text{ predrug latency}}{\text{Cutoff period }-\text{ predrug latency}}\;\times \;100$$

#### Tail immersion test

The procedure was carried out as previously reported (Janssen et al., 1963). The temperature of the water bath was set constant at 52 ± 1°C. Mice that exerted deflection of tail from warm water within 1.5–3.5 s were selected for the study. Mice were gently immobilized for 25–30 s using “Chux” and 1–2 cm of their tail was submersed into the warm water. The violent flick response of mice was taken as end point of nociception. A cut off period of 20 s was maintained to avoid injury to mice. The latency time at 30, 45, 60, 90, and 120 min following administration of vehicle, morphine (i.p.) or MEPS were recorded. A pre-treatment latency was obtained before administration. The same test was carried out with the prior administration of naloxone (i.p.) as described in hot plate test and % MPE was calculated from the latency periods.

#### Acetic acid-induced writhing test

The effect of MEPS against chemical induced central and peripheral nociception was evaluated by performing acetic acid-induced writhing test. To induce writhing, 0.7% acetic acid (10 mL/kg) was injected (i.p.) 30 min after the administration of vehicle or MEPS and 15 min of diclofenac sodium treatment. Mice were placed in box, left for 5 min and then the number of writhing was counted for the next 10 min (Vogel, 2007). The writhing was characterized by abdominal contraction, stretching or bending of the body, trunk and/or pelvis ending with limbs extension.

#### Formalin-induced licking test

Formalin was injected in the sub-plantar region of the right hind paw of mice to induce pain. Each mouse was injected with a volume of 20 μl of 1.35% formalin solution (0.5% formaldehyde) made up in saline. Formalin was injected 30 min after the administration of vehicle or MEPS. The standard drug morphine was injected 15 min before the formalin injection. Right hind paw licking of mice was measured from 0 to 5 min as early phase response (neurogenic phase) and from 15 to 25 min as late phase response (inflammatory phase). The licking was considered as indicative of nociception (Coelho et al., 2005).

#### Glutamate-induced paw licking and edema test

The mice were treated with vehicle, MEPS or diclofenac sodium first. The thickness of right hind paw of each mouse was measured at this stage using digital slide calipers. Glutamate was administered to the sub-plantar region of the right hind paw of each mouse (20 μL/20 μmol per paw) 30 min after the administration of vehicle or MEPS and 15 min after standard drug treatment. To avoid irritation, pH of the preparation was adjusted at 7.4. After glutamate challenge the mice were placed in observation chambers. The licking of injected paw was counted for 15 min and paw thickness was measured again for each mouse. The measure of paw thickness before glutamate injection was subtracted from paw thickness after glutamate challenge to determine the degree of edema (Δ) in mm (Rodrigues et al., 2012).

### Experimental procedures for neruopharmacological test

#### Open field test

Open field test was performed to evaluate effect of MEPS on locomotion of mice. The experiment was performed in an isolated, silent and dimly lit area. The dimension of apparatus of open field was 50 × 50 × 50 cm^3^ in size. The floor of the open field was divided into series of squares and each square were alternatively colored black and white. Each mouse was placed at the center of the field after the treatment with vehicle, diazepam or MEPS. Then the number of squares traveled was counted for 3 min at 0, 30, 60, 90, and 120 min (Gupta et al., 1971). The % inhibition was calculated by using the formula, where “MST” means mean squares traveled:

$$\begin{array}{l} \%\text{ inhibition}\\ =\;\frac{\text{MST by control group }-\text{ MST by treatment group}}{\text{MST by control group}}\;\times \;100 \end{array}$$

#### Hole cross test

The hole cross test was performed as previously describe by Takagi et al. (1971). A 30 × 20 × 14 cm^3^ size hole cross box was used to perform the experiment. A fixed partition into the middle of the box divided it into two compartments. There was a 3 cm hole to make easy passage for mice in the partition. Mice were treated with vehicle, diazepam or MEPS and immediately after treatment mice were placed beside the wall of one compartment facing to the hole. Then the number of movement from one compartment to other through the hole was counted for a period of 3 min at 0, 30, 60, 90, and 120 min. The % inhibition was calculated from the number of movements.

#### Sodium thiopental-induced sleeping time test

Mice were treated with vehicle, diazepam or MEPS. To induce sleep, sodium thiopental (40 mg/kg) was administered to each mouse 30 min after the treatments and placed in an observation chamber. After administration of sodium thiopental, time was counted till loss of righting reflex and recorded as the latent period. The duration of sleep was recorded from loss of righting reflex to recovery of righting reflex (Ferrini et al., 1974).

#### Elevated plus maze test

Elevated plus maze test was performed to evaluate the anxiolytic effect of MEPS. The maze consisted of two open arms (50 × 10 cm) and two enclosed arms (50 × 10 × 40 cm). The arms were arranged such a way that each type was opposite to each other and was elevated to 50 cm height above the floor. The experiment was performed in an isolated and silent area. Vehicle, MEPS or diazepam were administered 30 min before the experiment. Each mouse was placed individually in the center of the maze facing a closed arm. Entry of four paws in an arm was defined as arm entries (Pellow et al., 1985). Then the following parameter was observed and counted: (a) number of entries, (b) time spent into close and open arms during 5 min observation period. The percentage of time spent on open arm by each animal was calculated as follows:

$$\%\text{ of spent time }=\;\frac{\text{Open arm time}}{\text{Open arm time }+\text{ Closed arm time}}\;\times \;100$$

### Statistical analysis

The results were expressed as mean ± SEM (*n* = 5). To perform statistical analysis of the results, One-Way analysis of variance (ANOVA) followed by Dunnett's *post-hoc* or Bonferroni test was used as appropriate. All statistical analysis was performed by SPSS 19 software. Differences between groups were considered significant at the level of *p* < 0.05.

## Results

### Preliminary phytochemical screening

The presence of carbohydrates, reducing sugars, resins, tannins, proteins, saponins, falvonoids, alkaloids, and steroids in MEPS was found in the preliminary phytochemical screening.

### GC-MS analysis

**Figure 1:**
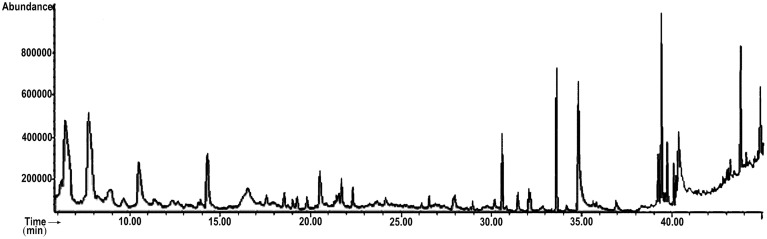
**Total ionic chromatogram (TIC) of methanol extract of ***P. sylvestris*** fruit (MEPS)**. TIC of MEPS obtained by GC-MS with energy of ionization of 70 eV.

The compounds identified by GC-MS analysis in MEPS are listed in Table 1 and the chromatogram is shown in Figure 1. Among 35, the major constituents were identified by comparing the relative percentage amount and they are: 2,3-Dihydro-3,5-dihydroxy-6-methyl-4H-pyran-4-one (14.24%), Catechol (13.49%), n-Hexadecanoic acid (9.25%), Methyl oleate (6.81%), Oleic acid (6.77%), 4-methylcatechol (5.92%), methyl palmitate (4.84%), 1-Ethynyl-7-(2'-methyl-1'-propenyl) tricycloheptanes (4.37%), Neophytadiene (4.03%), p-methoxyphenethyl alcohol (3.93%), Squalene (3.44%), Linoleate (3.09%), Hexadecanoic acid, 2-hydroxy-1-(hydroxymethyl) ethyl ester (2.87%), Lauric acid (2.50%), Phytol (2.05%), Ethanamine, N-ethyl-N-nitroso (1.17%), (-)-Loliolide (1.16%).

**Table 1:** **Compounds identified in MEPS by GC-MS analysis and their biological activity**.

**SL no**.	**RT(min)**	**% PA**	**Compound Name**	**Biological Activity**	**References**
01	6.191	1.71	Diethylnitrosamine	Not found	–
02	6.426	14.24	2, 3-Dihydro-3, 5-dihydroxy-6-methyl-4H-pyran-4-one	Antioxidant, autonomic nerve stimulator, anti proliferative and pro-apoptotic	Ban et al., 2007; Beppu et al., 2012; Yu et al., 2013
03	7.725	13.49	Catechol	Anti inflammatory	Zheng et al., 2008
04	10.488	5.92	4-methylcatechol	Analgesic and anti-depressant, brain derived neurotrophic factor stimulator, antioxidant	Hanaoka et al., 1994; Jongberg et al., 2011; Fukuhara et al., 2012
05	14.305	4.37	1-Ethynyl-7-(2'-methyl-1'-propenyl) tricyclo heptanes	Not found	–
06	16.502	3.99	p-methoxyphenethyl alcohol	Bacteriostatic	Khafagy and Lambooy, 1966
07	16.556	0.73	2,4-Di-tert-butylphenol	Antioxidant	Yoon et al., 2006
08	19.827	0.73	3, 4-dihydro-8-hydroxy-3-methyl-isocoumarin	Antioxidant, Fungicidal	Schulz et al., 1995; Liu et al., 2007
09	20.519	2.50	Lauric acid	Antimicrobial, anti inflammatory	Nakatsuji et al., 2009
10	21.732	1.38	Diethyl Phthalate	Not found	–
11	22.350	0.83	Cyclopentane, 3-heptyl-	Not found	–
12	26.550	0.41	Methyl tetradecanoate	Not found	–
13	27.969	1.16	(-)-Loliolide	Antidepressant, immunosuppressive	Neergaard et al., 2010
14	30.595	4.03	Neophytadiene	Antibacterial, anti inflammatory	Mustapa et al., 2015
15	32.157	0.77	Hexadecane	Not found	–
16	33.599	4.84	methyl palmitate	Anti inflammatory, antifibrotic	El-Demerdash, 2011
17	34.818	9.25	n-Hexadecanoic acid	Anti inflammatory	Aparna et al., 2012
18	39.247	3.09	Methyl linoleate	Not found	–
19	39.436	6.81	Methyl oleate	Not found	–
21	39.745	2.05	Phytol	Antinociceptive, antioxidant, anti inflammatory, anticonvulsant, sedative, anxiolytic	Costa et al., 2012, 2014; Santos et al., 2013; Silva et al., 2013
22	40.111	1.08	Methyl stearate	Not found	–
23	40.248	0.88	Linoleic acid	Anti inflammatory	Zhao et al., 2005
24	40.380	6.77	Oleic acid	Anti inflammatory	Zhao et al., 2005
25	42.852	0.31	2-Butenenitrile, 2-chloro-3- (4-methoxyphenyl)-	Not found	–
26	43.035	0.37	2 (3H)- Furanone, dihydro-5-tetradecyl-	Not found	–
27	43.138	0.24	Methyl eicosanoate	Not found	–
28	43.252	0.41	Phosphine, chloromenthylphenyl-	Not found	–
29	43.813	3.44	Squalene	Antioxidant	Kim and Karadeniz, 2012
30	44.134	0.29	4-Dehydroxy-N- (4, 5-methylenedioxy-2-nitrobenzylidene) tyramine	Not found	–
31	44.597	0.59	Phenanthridinium, 5, 6-dimethyl-, iodide	Not found	–
32	44.763	0.33	-Cheilanth-12-enic Methyl Ester	Not found	–
33	44.843	0.33	Adamantane, 1-isothiocyanato-3-methyl	Not found	
34	44.900	2.87	Hexadecanoic acid, 2-hydroxy-1-(hydroxymethyl) ethyl ester	Not found	–
35	45.066	0.65	Methyl docosanoate	Not found	–

*SL, serial; RT, retention time; PA, peak area*.

### Acute toxicity

Oral administration of MEPS did not produce any toxicity at doses of 500–6000 mg/kg. During the 72 h observation period no behavioral changes, allergic reaction or mortality were observed. This result demonstrates that MEPS have lower toxicity profile.

### Hot plate test

Oral administration of MEPS demonstrated significant increase in latency period to thermal stimulus at the doses of 300 and 450 mg/kg in hot plate test (*p* < 0.01) as shown in Table 2. The protection against heat-induced pain response of MEPS was dose-dependent and stronger at 450 mg/kg dose than other doses. The reaction time for standard drug morphine was more than any dose of MEPS at all observation period. MEPS also showed significant % MPE at all experimental doses (*p* < 0.05). The antinoceptive activity of MEPS at all four doses and of morphine was significantly antagonized by naloxone (*p* < 0.05); (Table 2).

**Table 2:** **Effect of MEPS, morphine and reversal effect of naloxone in hot plate test**.

**Treatment**	**Dose (mg/kg)**	**Latency Period (sec) (%MPE)**					
		**Pre-treatment**	**30 min**	**45 min**	**60 min**	**90 min**	**120 min**
Vehicle	–	3.32 ± 0.41	3.64 ± 0.39	3.71 ± 0.30	3.74 ± 0.42	3.82 ± 0.33	3.65 ± 0.40
Morphine	5 (i.p.)	3.02 ± 0.21	7.56 ± 0.75^**^ (26.80)	9.88 ± 0.39^**^ (40.22)	11.23 ± 0.77^**^ (48.36)	7.85 ± 0.38^**^ (28.39)	6.41 ± 0.51^**^ (19.81)
MEPS	50	3.20 ± 0.21	4.29 ± 0.27 (6.49)	5.02 ± 0.25 (10.75)	5.73 ± 0.40 (14.98)	5.42 ± 0.30 (13.13)	4.49 ± 0.35 (7.61)
MEPS	150	3.42 ± 0.30	5.17 ± 0.59 (10.59)	5.39 ± 0.45 (11.97)	6.05 ± 0.62 (16.01)	5.93 ± 0.97 (14.96)	5.57 ± 0.44 (12.81)
MEPS	300	3.38 ± 0.35	5.19 ± 0.44 (10.68)	5.53 ± 0.39 (12.71)	6.51 ± 0.40^*^ (18.57)	7.72 ± 0.34^**^ (26.01)	6.60 ± 0.28^**^ (19.23)
MEPS	450	3.33 ± 0.34	6.24 ± 0.17^*^ (17.28)	6.48 ± 0.47^**^ (18.70)	7.38 ± 0.69^**^ (24.00)	7.73 ± 0.54^**^ (26.37)	8.88 ± 0.55^**^ (33.37)
NLX	2 (i.p.)	3.21 ± 0.22	2.89 ± 0.16	2.74 ± 0.15	2.61 ± 0.16	2.81 ± 0.18	2.95 ± 0.28
NLX + Morphine	2+5	3.23 ± 0.11	3.58 ± 0.12^a^ (2.12)	3.24 ± 0.18^a^ (0.09)	3.05 ± 0.22^a^ (−1.05)	2.80 ± 0.14^a^ (−2.54)	3.07 ± 0.15^a^ (−0.92)
NLX + MEPS	2+50	3.28 ± 0.20	3.51 ± 0.17 (1.33)	3.11 ± 0.28^b^ (−1.03)	3.74 ± 0.34^b^ (2.71)	4.08 ± 0.40^b^ (4.74)	3.23 ± 0.47 (−0.29)
NLX + MEPS	2+150	3.24 ± 0.17	3.72 ± 0.22 (2.87)	3.15 ± 0.18^c^ (−0.57)	3.79 ± 0.30^c^ (3.28)	3.53 ± 0.22^c^ (1.70)	3.19 ± 0.24^c^ (−0.32)
NLX + MEPS	2+300	3.25 ± 0.34	3.81 ± 0.33 (3.39)	3.40 ± 0.22^d^ (0.86)	4.53 ± 0.50^d^ (7.75)	4.95 ± 0.5^d^ (10.27)	4.24 ± 0.45^d^ (5.95)
NLX + MEPS	2+450	3.28 ± 0.20	3.93 ± 0.24^e^ (3.83)	4.17 ± 0.3^e^ (5.31)	4.55 ± 0.39^e^ (7.60)	3.39 ± 0.29^e^ (0.64)	3.25 ± 0.30^e^ (−0.21)

*Each value is presented as mean ± SEM (n = 5), MEPS, Methanol extract of P. sylvestris fruit pulp; NLX, Naloxone*.

* *p < 0.01*,

** *p < 0.001 compared with the control group (Dunnett's test)*.

a *p < 0.001*,

b *p < 0.05*,

c *p < 0.05*,

d *p < 0.01*,

e *p < 0.01 compared with the morphine, MEPS 50, MEPS 150, MEPS 300, MEPS 450 group respectively (Bonferroni's test)*.

### Tail immersion test

MEPS significantly reduced the hot-water induced nociception at all experimental doses in tail immersion test (*p* < 0.001). The antinociceptive effect of MEPS and morphine are shown in Table 3. Both MEPS and morphine significantly increased the latency time. The % MPE of the extract was also significant at all experimental doses (*p* < 0.05). The % MPE value for morphine was higher than MEPS at all observation periods. The activity of MEPS against thermal nociception was dose-dependent. Naloxone significantly reversed the antinoceptive effect of morphine and of MEPS at all experimental doses (*P* < 0.01).0.1.

**Table 3:** **Effect of MEPS, morphine and reversal effect of naloxone in tail immersion test**.

**Treatment**	**Dose (mg/kg)**	**Latency Period (s) (%MPE)**					
		**Pre-treatment**	**30 min**	**45 min**	**60 min**	**90 min**	**120 min**
Vehicle	–	2.25 ± 0.11	2.55 ± 0.19	2.42 ± 0.11	2.56 ± 0.15	2.85 ± 0.31	2.86 ± 0.33
Morphine	5 (i.p.)	2.37 ± 0.15	8.88 ± 0.19^*^ (36.95)	9.43 ± 0.18^*^ (40.02)	10.60 ± 0.36^*^ (46.70)	8.35 ± 0.19^*^ (33.87)	4.34 ± 0.18^*^ (11.09)
MEPS	50	2.25 ± 0.19	2.81 ± 0.11 (3.16)	3.47 ± 0.12^*^ (6.83)	3.84 ± 0.11^*^ (8.93)	3.99 ± 0.17 (9.83)	3.45 ± 0.14 (6.75)
MEPS	150	2.49 ± 0.15	3.51 ± 0.12 (5.73)	4.04 ± 0.18^*^ (8.78)	4.39 ± 0.18^*^ (10.78)	4.90 ± 0.12^*^ (13.74)	3.98 ± 0.14 (8.48)
MEPS	300	2.74 ± 0.19	3.79±0.15^*^ (6.09)	4.54 ± 0.12^*^ (10.37)	4.92 ± 0.12^*^ (12.58)	5.47 ± 0.12^*^ (15.84)	4.46 ± 0.14^*^ (9.93)
MEPS	450	2.56 ± 0.12	4.35 ± 0.13^*^ (10.24)	4.80 ± 0.16^*^ (12.83)	5.34 ± 0.14^*^ (15.96)	5.68 ± 0.13^*^ (17.90)	5.96 ± 0.12^*^ (19.46)
NLX	2 (i.p.)	2.58 ± 0.15	2.49 ± 0.19	2.48 ± 0.18	2.33 ± 0.17	2.54 ± 0.22	2.33 ± 0.18
NLX + Morphine	2+5	2.69 ± 0.18	2.86 ± 0.12^a^ (0.96)	2.55 ± 0.11^a^ (−0.83)	2.41 ± 0.14^a^ (−1.64)	2.60 ± 0.20^a^ (−0.55)	2.55 ± 0.18^a^ (−0.85)
NLX + MEPS	2+50	2.58 ± 0.13	3.04 ± 0.11 (2.48)	2.43 ± 0.16^b^ (−1.04)	2.39 ± 0.13^b^ (−1.22)	2.57 ± 0.14^b^ (−0.25)	3.27 ± 0.26 (3.29)
NLX + MEPS	2+150	2.74 ± 0.25	2.34 ± 0.12^c^ (−2.71)	2.52 ± 0.16^c^ (−1.64)	2.62 ± 0.19^c^ (−1.14)	2.69 ± 0.20^c^ (−0.73)	2.78 ± 0.15^c^ (0.52)
NLX + MEPS	2+300	2.71 ± 0.17	2.60 ± 0.28^d^ (−0.72)	2.42 ± 0.11^d^ (−1.72)	2.60 ± 0.17^d^ (−0.67)	2.91 ± 0.19^d^ (1.11)	2.58 ± 0.21^d^ (−0.79)
NLX + MEPS	2+450	2.76 ± 0.23	2.44 ± 0.14^e^ (−1.88)	2.67 ± 0.32^e^ (−0.53)	2.79 ± 0.34^e^ (0.22)	2.58 ± 0.13^e^ (−1.15)	2.99 ± 0.30^e^ (1.32)

*Each value is presented as mean ± SEM (n = 5), MEPS = Methanol extract of P. sylvestris fruit pulp; NLX = Naloxone*.

* *p < 0.001 compared with the control group (Dunnett's test)*.

a *p < 0.001*,

b *p < 0.01*,

c *p < 0.01*,

d *p < 0.01*,

e *p < 0.001 compared with the morphine, MEPS 50, MEPS 150, MEPS 300, MEPS 450 group respectively (Bonferroni's test)*.

### Acetic acid-induced writhing

As shown in Table 4, acetic acid-induced writhing response was significantly decreased by standard drug (diclofenac sodium) and MEPS at all doses (*p* < 0.001). The % inhibition of writhing by MEPS was dose-dependent and highest at the dose of 450 mg/kg (63.34%). The standard drug (diclofenac sodium) showed maximum % inhibition (80.05%).

**Table 4:** **Effect of MEPS and diclofenac sodium in the acetic acid-induced writhing test**.

**Treatment**	**Dose (mg/kg)**	**Number of Writhing**	**% Inhibition**
Vehicle	–	34.1 ± 0.62	–
Diclofenac sodium	10 (i.p.)	6.80 ± 0.25^*^	80.05
MEPS	50	24.30 ± 1.10^*^	28.74
MEPS	150	21.20 ± 1.49^*^	37.83
MEPS	300	16.70 ± 0.97^*^	51.02
MEPS	450	12.50 ± 0.76^*^	63.34

*Each value is presented as mean ± SEM (n = 5), MEPS, Methanol extract of P. sylvestris fruit pulp*.

* *p < 0.001 compared with the control group (Dunnett's Test)*.

### Formalin-induced licking

MEPS at the doses of 150, 300, and 450 mg/kg and the centrally acting analgesic, morphine, produced significant inhibition of formalin-induced licking (*p* < 0.001) in both early and late phase of formalin-induced licking test (Table 5). The inhibition of licking by MEPS was dose-dependent and percent of inhibition was highest at 450 mg/kg in both early and late phases (68.21% and 75.68% respectively). Morphine completely inhibited the nociceptive effect at late phase.

**Table 5:** **Effect of MEPS and morphine in formalin-induced paw licking test**.

**Treatment**	**Dose (mg/kg)**	**Licking time (sec) of the hind paw**			
		**Early phase (0–5 min)**	**% inhibition**	**Late phase (15–25 min)**	**% inhibition**
Vehicle	–	132.13 ± 4.45	–	153.16 ± 4.71	–
Morphine	5 (i.p.)	36.73 ± 5.09^*^	72.20	0.00 ± 0.00^*^	100.00
MEPS	50	110.76 ± 5.99^**^	16.18	109.06 ± 5.81^*^	28.79
MEPS	150	75.99 ± 6.57^*^	42.49	69.99 ± 5.70^*^	54.30
MEPS	300	59.18 ± 5.13^*^	55.21	54.16 ± 3.22^*^	64.64
MEPS	450	42.01 ± 5.15^*^	68.21	37.24 ± 3.39^*^	75.68

*Each value is presented as mean ± SEM (n = 5), MEPS, Methanol extract of P. sylvestris fruit pulp*.

* *p < 0.001*,

** *p < 0.05 compared with the control group (Dunnett's Test)*.

### Glutamate-induced paw licking and edema

MEPS significantly reduced the glutamate-induced paw licking at the doses of 150, 300, and 450 mg/kg (*p* < 0.001). The inhibitory effect of MEPS was dose-dependent and highest at the dose of 450 mg/kg (47.71%). However, the standard drug (diclofenac sodium) showed maximum inhibition (71.06%). The extract also showed significant reduction of paw edema (*p* < 0.001) at all the test doses (Table 6).

**Table 6:** **Effect of MEPS and diclofenac sodium in glutamate-induced paw licking and edema test**.

**Treatment**	**Dose (mg/kg)**	**Licking time (s)**	**% Inhibition**	**(Δ) Paw thickness (mm)**	**% Inhibition**
Vehicle	–	205.74 ± 3.73	–	1.38 ± 0.04	–
Diclofenac sodium	10 (i.p.)	59.54 ± 4.14^*^	71.06	0.49 ± 0.06^*^	64.24
MEPS	50	186.32 ± 3.85^**^	9.43	0.87 ± 0.07^*^	36.77
MEPS	150	147.83 ± 3.43^*^	28.14	0.85 ± 0.04^*^	38.23
MEPS	300	119.47 ± 6.73^*^	41.93	0.79 ± 0.02^*^	42.15
MEPS	450	107.58 ± 3.39^*^	47.71	0.73 ± 0.05^*^	46.66

*Each value is presented as mean ± SEM (n = 5), MEPS, Methanol extract of P. sylvestris fruit pulp*.

* *p < 0.001*,

** *p < 0.05 compared with the control group (Dunnett's Test)*.

### Open field test

MEPS significantly inhibited the locomotor activity at the doses of 50 and 150 mg/kg for first 60 and 90 min respectively. However, at the doses of 300 and 450 mg/kg, MEPS significantly reduced the locomotor activity from 30 to 120 min like the reference drug diazepam (*p* < 0.05). The percent inhibition of the dose 450 mg/kg was close to that of diazepam at 30 and 60 min (Table 7).

**Table 7:** **Effect of MEPS and diazepam in open field test**.

**Treatment**	**Dose (mg/kg)**	**Number of square crossed (% inhibition)**				
		**0 min**	**30 min**	**60 min**	**90 min**	**120 min**
Vehicle	–	85.40 ± 2.94	68.20 ± 4.05	56.40 ± 2.67	38.80 ± 2.46	33.20 ± 2.59
Diazepam	1 (i.p.)	84.40 ± 2.16	35.40 ± 1.86^*^ (48.09)	28.80 ± 3.29^*^ (48.93)	17.60 ± 3.34^*^ (54.64)	18.40 ± 1.16^*^ (44.58)
MEPS	50	86.80 ± 5.50	50.80 ± 4.24^*^ (25.51)	37.40 ± 2.38^*^ (33.69)	32.80 ± 1.56 (15.46)	27.20 ± 1.15 (18.07)
MEPS	150	88.20 ± 5.24	41.80 ± 2.46^*^ (38.71)	33.00 ± 3.86^*^ (41.49)	24.40 ± 2.50^*^ (37.11)	24.80 ± 2.72 (25.30)
MEPS	300	86.60 ± 3.93	38.40 ± 1.96^*^ (43.69)	30.20 ± 2.15^*^ (46.45)	23.40 ± 1.29^*^ (39.69)	22.80 ± 4.05^*^ (31.32)
MEPS	450	88.80 ± 5.95	36.80 ± 2.92^*^ (46.04)	28.20 ± 2.08^*^ (50.00)	18.40 ± 1.16^*^ (52.58)	15.40 ± 1.43^*^ (53.61)

*Each value is presented as mean ± SEM (n = 5), MEPS, Methanol extract of P. sylvestris fruit pulp*.

* *p < 0.05 compared with the control group (Dunnett's Test)*.

### Hole cross test

MEPS demonstrated significant inhibition of movement at the doses of 300 and 450 mg/kg throughout the observation periods in hole cross test (*p* < 0.05). On the other hand the dose 50 and 150 mg/kg showed significant effect at later observation periods. The standard drug diazepam showed significant (*p* < 0.05) reduction of movement and highest percent inhibition of locomotion at all the study periods. The reduction of locomotor activity by MEPS was dose-dependent (Table 8).

**Table 8:** **Effect of MEPS and diazepam in hole cross test**.

**Treatment**	**Dose (mg/kg)**	**Number of hole crossed (% inhibition)**				
		**0 min**	**30 min**	**60 min**	**90 min**	**120 min**
Vehicle	–	14.20 ± 0.58	11.00 ± 0.95	9.00 ± 0.54	8.60 ± 0.68	8.00 ± 0.45
Diazepam	1 (i.p.)	13.80 ± 0.86	5.40 ± 0.68^*^ (50.91)	2.80 ± 0.37^*^ (68.89)	2.00 ± 0.44^*^ (76.74)	1.40 ± 0.24^*^ (82.50)
MEPS	50	13.20 ± 0.86	9.60 ± 0.98 (12.73)	7.20 ± 0.73 (20.00)	6.40 ± 0.81^*^ (25.58)	5.80 ± 0.73^*^ (27.50)
MEPS	150	14.40 ± 1.07	9.40 ± 0.51 (14.74)	6.40 ± 0.51^*^ (28.89)	4.80 ± 0.58 (44.19)	4.40 ± 0.51^*^ (45.00)
MEPS	300	13.20 ± 0.91	8.00 ± 0.32^*^ (27.27)	5.20 ± 0.37^*^ (42.22)	4.00 ± 0.32^*^ (53.49)	3.40 ± 0.51^*^ (57.50)
MEPS	450	13.40 ± 0.74	7.20 ± 0.49^*^ (34.54)	4.80 ± 0.66^*^ (46.67)	3.20 ± 0.58^*^ (62.79)	2.60 ± 0.68^*^ (67.75)

*Each value is presented as mean ± SEM (n = 5), MEPS, Methanol extract of P. sylvestris fruit pulp*.

* *p < 0.05 compared with the control group (Dunnett's Test)*.

### Sodium thiopental-induced sleeping time test

The doses 150, 300, and 450 mg/kg of MEPS showed significant (*p* < 0.001) reduction in onset of sleep and increased sleep duration. The dose 50 mg/kg also increased the sleeping time and decreased onset of sleep (*p* < 0.05). The dose-dependent effect of MEPS was clear from the observations of 50–450 mg/kg doses. The effect of diazepam was found most significant considering the onset of sleeping and highest duration of sleeping (*p* < 0.001) (Table 9).

**Table 9:** **Effect of MEPS and diazepam in sodium thiopental-induced sleeping time test**.

**Treatment**	**Dose (mg/kg)**	**Onset of Sleep (s)**	**Duration of Sleep (min)**
Vehicle + ST	–	190.94 ± 1.27	32.09 ± 1.14
Diazepam + ST	1+40	93.93 ± 3.05^*^	96.34 ± 2.09^*^
MEPS + ST	50+40	174.31 ± 4.49^**^	40.01 ± 1.11^**^
MEPS + ST	150+40	156.6 ± 2.31^*^	50.67 ± 1.30^*^
MEPS + ST	300+40	103.83 ± 5.74^*^	71.30 ± 1.81^*^
MEPS + ST	450+40	92.15 ± 5.02^*^	88.73 ± 4.48^*^

*Each value is presented as mean ± SEM (n = 5), MEPS, Methanol extract of P. sylvestris fruit pulp; ST, sodium thiopental*.

* *p < 0.001*,

** *p < 0.05 compared with the control group (Dunnett's Test)*.

### Elevated plus maze test

Table 10 clearly shows that the time spent in open arm was increased and the percent of time spent was significant (*p* < 0.05) at all the doses of MEPS in elevated plus maze test. The standard drug diazepam also significantly increased the percent time spent in open arm (*p* < 0.05). The percent open arm entries of MEPS at the doses of 150, 300, and 450 mg/kg and of diazepam were significant (*p* < 0.05). In addition, total number of entries was also reduced significantly by the extract and diazepam compared to the control group.

**Table 10:** **Effect of MEPS and diazepam in elevated plus maze test**.

**Treatment**	**Dose (mg/kg)**	**Open arm time (s)**	**% Open arm time (s)**	**Open arm entries**	**% open arm entries**	**Total arm entries**
Vehicle	–	39.05±5.92	16.31±2.40	6.20±1.39	31.81±3.46	22±2.34
Diazepam	1	164.98±6.50	64.25±1.43^*^	10.80±1.49	67.58±2.09^*^	12.6±1.21^*^
MEPS	50	100.32±6.01	40.12±1.76^*^	6.80±1.31	37.07±6.62	16.4±1.66
MEPS	150	106.83±6.32	43.56±1.83^*^	8.00±1.40	53.48±5.98^**^	15.4±0.93^**^
MEPS	300	138.87±14.72	52.02±5.47^*^	9.20±1.35	55.27±5.91^*^	15.8±0.80^**^
MEPS	450	136.62±18.39	54.43±6.21^*^	11.4±1.75	63.48±4.70^*^	15±1.51^**^

*Each value is presented as mean ± SEM (n = 5), MEPS, Methanol Extract of P. sylvestris fruit pulp*.

* *p < 0.01*,

** *p < 0.05 compared with the control group (Dunnett's Test)*.

## Discussion

The results of the present study indicate peripheral and central antinociceptive, CNS depressant, sedative and anxiolytic effect of MEPS at different doses in mice models. In addition, study of acute toxicity at the doses of 500–6000 mg/kg suggests that MEPS possess low toxicity profile.

The significant (*p* < 0.01) inhibition of the thermally induced nociception and increase of latency time in hot plate test demonstrates the central antinociceptive activity of MEPS (Table 2). The effect was also observed (*p* < 0.001) in the tail immersion test (Table 3). The tail withdrawal response is selective only for centrally acting analgesics (Srinivasan et al., 2003). Therefore, the outcome of tail immersion test supports the effect of MEPS in hot plate test. The hot plate test induces nociception via supraspinal reflex whereas tail immersion test induce nociceptive pain by spinal reflexes (Chapman et al., 1985). Opioid agents exhibit their analgesic effect via both supraspinal (μ_1_, κ_3_, δ_1_, σ_2_) and spinal (μ_2_, κ_1_, δ_2_) receptors (Hosseinzadeh et al., 2002; Jinsmaa et al., 2004, 2005). Therefore the antinociceptive activity of MEPS is due to action on spinal and supraspinal receptors. Naloxone significantly antagonized (*p* < 0.05) the antinociceptive effect of morphine and MEPS at different doses level in both thermal pain models. This confirms the opioid receptors mediated antinociceptive activity of MEPS.

Acetic acid-induced writhing test is a commonly used method to evaluate the antinociceptive activity of peripheral and central analgesic agents. The action of acetic acid causes the release of endogenous histamine, prostaglandins (PGs), serotonin, bradykinin, cycloxoygenase (COX), lipoxygenase (LOX), and cytokines (TNF-α, IL-1β, and IL-8). These visceral inflammatory mediators enter the dorsal horn of central nervous system and stimulate primary afferent nociceptors (Ikeda et al., 2001) and results in induction of pain expressing abdominal constriction (Bley et al., 1998). Oral administration of MEPS (*p* < 0.001) significantly reduced the number of abdominal constriction induced by acetic acid in mice (Table 4). The result clearly indicates that the antinociception produced by MEPS is due to its inhibition of COX, LOX and other endogenous inflammatory mediators as well as of primary afferent nociceptors signal transduction. The identified compound phytol has been reported to give antinociceptive activity via central and peripheral mechanisms (Santos et al., 2013). Besides histamine, serotonin, bradykinin, PGE_2_ and cytokines inhibitory activity of phytol (Silva et al., 2013) provide justification of the result.

The formalin-induced nociception is mediated via direct stimulation of sensory afferent fibers by the chemical nociceptors especially C-fibers in early phase and peripheral inflammatory mediators such as histamine, prostaglandins (PGs), serotonin and bradykinin in late phase (Le Bars et al., 2001; Parada et al., 2001). In formalin-induced licking test peripherally acting analgesics inhibit early phase nociception where the centrally acting analgesics inhibit the nociception of both phases (Hunskaar and Hole, 1987; Tjølsen et al., 1992). The results showed that MEPS significantly (*p* < 0.001) reduced paw licking at both early and late phases in a dose-dependent manner. The effect was stronger in late phase (Table 5). Therefore the inhibition of nociception by MEPS in both phases gives further evidence of its central antinociceptive effect. Moreover, the late phase inhibition suggests the anti-inflammatory potential of MEPS.

MEPS produced significant dose-dependent inhibition (*p* < 0.001) in both glutamate-induced paw licking and edema (Table 6). The glutamate induced nociceptive response and edema formation involves (N-methyl-D-aspartate) NMDA and non-NMDA (AMPA, Kainate) receptors respectively in peripheral, spinal and supraspinal sites of action. Glutamate causes neuropeptides (substance P) and pro-inflammatory cytokines (TNF-α, IL-1β) release to transmit nociceptive signals from the peripheral nervous system to the dorsal horn of the spinal cord. Moreover, the pro-inflammatory signals are mediated by, ROS (Reactive oxygen species) and NOS (Nitric oxide synthase) participated stimulation of TNF-α, IL-1, and IL-6 genes through the activation of the redox-sensitive transcription factor NF-κB (Beauparlant and Hiscott, 1996; Beirith et al., 2002; Ribas et al., 2008). Therefore, from the results it can be suggested that MEPS inhibited the release of neuropeptides and activation of NMDA and non-NMDA receptors as well as interrupted the ROS and NOS mediated pro-inflammatory signals. The compounds such as 2,3-Dihydro-3,5-dihydroxy-6-methyl-4H-pyran-4-one, catechol, oleic acid and linoleic acids found in MEPS has been reported to inhibit the NF-κB, NOS, TNF-α, and TNF-α gene expression respectively (Zhao et al., 2005; Ban et al., 2007; Zheng et al., 2008) which strongly suggests the pro-inflammatory signal inhibitory activity of MEPS.

We have detected the presence of carbohydrates, steroids, alkaloids, saponins, and tannins by preliminary phytochemical screening and considerable amount of phenolic content (128.6 ± 2.7 mg_GAE_/g) has been found in *P. sylvestris* fruit (Prakash et al., 2013). Scientific investigations reported that plant materials containing flavonoids, phenols and tannins are responsible for analgesic and CNS activity (Takahashi et al., 1986; Starec et al., 1988; Mills and Bone, 2000; Morteza-Semnani et al., 2006).

Beside antinociceptive activity, MEPS has also been found to have neurobehavioral effects. Both in open field and hole cross test MEPS depressed the central nervous system. The locomotor activity of mice gradually decreased during the period of observation (Tables 7, 8). In addition, MEPS significantly decreased latency time and increased the sleeping time in sodium thiopental-induced sleeping time test (Table 9) suggesting its possible sedative effect. CNS depressant and sedative drugs potentiate the gamma aminobutyric acid (GABA) mediated post synaptic inhibition via allosteric modification of GABA receptors as well as decrease the sleep latency and increase sleeping time (Sieghart and Sperk, 2002; Aladeokin and Umukoro, 2011; O'Donnell and Shelton, 2011). Therefore the significant decrease of locomotion and sleep enhancement activity of MEPS reveals its potential action on GABA receptors. The GABA_A_ receptor mediated sedative activity of identified compound phytol (Costa et al., 2014) provides further evidence of GABAergic mechanism of action of MEPS.

Another potentiality of MEPS was revealed in elevated plus maze test. This test is frequently used to evaluate the anxiolytic or anxiogenic properties of drug. The fact is rodents are extremely aversive to an open area and the major index of anxiety is the frequency and time spent in the open arms (Pellow and File, 1986). The benzodiazepine drugs exert their anxiolytic activity via their action on GABA/benzodiazepine/chloride (GBC) complex mechanism and increase the percent of spent time, frequency of entries in open arm and also reduce total number of entries (Pellow et al., 1985; Stephens et al., 1986). The results showed that diazepam and MEPS significantly (*p* < 0.05) increased percentage of spent time, frequency of entries in open arm and reduced total arm entries in elevated plus maze (Table 10). The findings of probable GABAergic-like mechanism of MEPS and action like reference anxiolytic drug diazepam may be a potential indicator of its anxiolytic-like activity. In addition the anxiolytic effect of phytol (Costa et al., 2014) found in MEPS support our results. Therefore the outcomes of our investigation rationalize the traditional implication of *P. sylvestris* fruit as nervine tonic in the treatment of CNS disorder like nervous debility.

From the results of different pain models it may be suggested that crude methanol extract *of P. sylvestris* fruit possesses potent and long-lasting antinociceptive activity. Inhibition of MEPS-induced antinociception by selective opioid antagonist, naloxone, suggests the involvement of opioid receptor mediated analgesia. The results also provide the evidence of inhibition of peripheral inflammatory mediators such as COX, LOX, PGs in pain inhibition. In addition the neuropharmacological evaluation also suggests the sleep enhancing and anxiolytic potentiality of *P. sylvestris* fruit which is possibly mediated via GABAergic pathway. These outcomes rationalize the use of *P. sylvestris* fruit in traditional medicine in painful conditions such as toothache, headache, arthritis backache or pain in buttocks, and also to induce sedation, as nervine tonic and in the treatment of nervous debility. The GC-MS analysis shows there are potential biologically active compounds present in *P. sylvestris* fruit. Taking the findings into account, it seems quite possible that *P. sylvestris* fruit may lead to the development of new natural products having analgesic and neuropharmacological effect.

## Author contributions

MZI, TA, and MS conceived and designed the study. MS performed the experiments and collected data. Data analysis was performed by MZI and MS. MS, SA, and MZI drafted the manuscript and all authors revised and approved the content of manuscript.

### Conflict of interest statement

The authors declare that the research was conducted in the absence of any commercial or financial relationships that could be construed as a potential conflict of interest.

## Abbreviations

MEPS: Methanol extract of *Phoenix sylvestris* fruit pulp
CNS: central nervous system
GC-MS: Gas chromatography-mass spectroscopy
ACE: Angiotensin I-converting enzyme
r.p.m: Rotation per minute
AVMA: American Veterinary Medical Association
icddr,b: International Center for Diarrhoeal Disease Research, Bangladesh
p.o: per oral
i.p.: intraperitoneal
NIST-MS: National Institute of Standards and Technology- mass spectroscopy
EI: electron ionization
TIC: total ionic chromatogram
% MPE: percentage of maximal possible effect
MST: mean square traveled by animals
ANOVA: analysis of variance
LD_50_: median lethal dose
COX: cycloxoygenase
LOX: lipoxygenase
TNF-α: Tumor necrosis factor alpha
IL-1β: Interleukin-1 beta
IL-8: Interleukin 8
PGs: Prostaglandins
NMDA: N-methyl-D-aspartate
AMPA: Alpha-amino-3-hydroxy-5-methylisoxazole-4-propionic acid
NF-κB: nuclear factor kappa-light-chain-enhancer of activated B cells
ROS: Reactive oxygen species
NOS: Nitric oxide synthase
GAE: Gallic acid equivalence method
GABA: gamma amino butyric acid
GBC: GABA/benzodiazepine/chloride complex
NLX: naloxone
ST: sodium thiopental
n: number of mice
RT: retention time
PA: peak area
eV: electron volt.
